# Generative AI–Powered Mental Wellness Chatbot for College Student Mental Wellness: Open Trial

**DOI:** 10.2196/71923

**Published:** 2025-07-28

**Authors:** Jazmin A Reyes-Portillo, Amy So, Kelsey McAlister, Christine Nicodemus, Ashleigh Golden, Colleen Jacobson, Jennifer Huberty

**Affiliations:** 1 Montclair State University, 1 Normal Ave, Montclair, NJ, 07043-1624, United States, 1 9736557945; 2 Fit Minded, Phoenix, AZ, United States; 3 Wayhaven, Charlotte, NC, United States; 4 Continuum Psychiatric Services, LLP, New York, NY, United States

**Keywords:** artificial intelligence, chatbots, anxiety, depression, college students

## Abstract

**Background:**

Colleges have turned to digital mental health interventions to meet the increasing mental health treatment needs of their students. Among these, chatbots stand out as artificial intelligence–driven tools capable of engaging in human-like conversations that have demonstrated some effectiveness in reducing depression and anxiety symptoms.

**Objective:**

This study aimed to assess the feasibility and acceptability of using Wayhaven, an artificial intelligence chatbot, among college students with elevated depression or anxiety symptoms. We also aimed to examine the preliminary effectiveness of Wayhaven in improving symptoms of anxiety and depression, hopelessness, agency, and self-efficacy among students.

**Methods:**

Participants were 50 racially and ethnically diverse college students with elevated depression or anxiety symptoms (n=45, 80% female; mean age 22.12*,* SD 4.42 years). Students were asked to use Wayhaven over the course of 1 week and completed assessments at preintervention, after 1 session, and 1 week.

**Results:**

Wayhaven use was associated with a significant decrease in depression (β=−1.62; *P*<.001), anxiety (β=−2.15; *P*<.001), and hopelessness (β=−.64; *P*<.001) and a significant increase in agency (β=.64; *P*=.32), self-efficacy (β=.53; *P*=.02), and well-being (*t*_40_=2.90; *P*=.006; *d*=0.45) across the study period. Most students also reported being satisfied with Wayhaven and it being a tool they would recommend to their peers.

**Conclusions:**

Findings suggest that Wayhaven may be a viable mental wellness resource for diverse students with elevated depression or anxiety symptoms.

## Introduction

Rates of depression, anxiety, and suicidal ideation have surged among college students since the COVID-19 pandemic [1,2]. From 2013 to 2021, the past-year prevalence rate of depression, anxiety, and suicidal ideation has increased by 134.6%, 109.5%, and 64%, respectively, among college students [3]. More than ever before, college counseling centers are unable to meet the increasing mental health needs of their students [4,5]. Students of color, in particular, have higher unmet treatment needs than their White peers [3]. At the same time, many who would benefit from services never seek treatment. Among college students, lack of perceived need for treatment, limited time, preference for self-help, and fear of stigma impede help-seeking [6,7].

To help address these issues, colleges have turned to digital mental health interventions (DMHIs). DMHIs refer to a range of health information technologies (eg, websites, mobile apps, and chatbots) designed to intervene in health conditions by changing behaviors, cognitions, and emotional states [8]. Among these, chatbots stand out as artificial intelligence (AI)–driven tools capable of engaging in human-like conversations. Chatbots can help monitor a patient’s progress, track symptoms and behaviors, facilitate the transfer of therapeutic content into their daily lives, and provide personalized support by delivering additional mental health resources [9]. Beyond offering convenient access to resources, AI-powered DMHIs are well-liked by college-aged individuals [10]. Features like active listening for rapport building and problem exploration through AI have been found to be not only acceptable but, in some cases, even preferred over human-to-human interactions [11]. This user-centered focus of DMHIs appears to be a key factor in their appeal to college-aged individuals [12].

Chatbots, such as Woebot and Tess, have been used to deliver cognitive behavioral therapy (CBT)–based self-help content in a text-based conversational format to college students experiencing depression and anxiety, demonstrating acceptability and some effectiveness in reducing depression and anxiety symptoms compared to psychoeducational control conditions [13,14]. While these tools use user-centered approaches, such as mood monitoring, psychoeducation, and validation, their interactions rely heavily on preprogrammed scripts and structured conversation pathways. This limits their ability to adapt dynamically to the nuanced, real-time needs of users or incorporate personalized context, such as past conversations or external user-specific data (eg, demographics).

To address these gaps, Wayhaven, a generative AI-powered mental wellness chatbot, delivers dynamic, context-aware mental well-being support tailored to each user’s unique needs. Its AI mental wellness coaches deliver personalized, context-aware support by incorporating user demographics, university-specific resources, and a tailored AI coach personality. Unlike scripted chatbots, Wayhaven dynamically adapts to users’ real-time needs while ensuring safety through advanced crisis detection and rerouting. This innovative approach positions Wayhaven as a unique tool for college students with elevated anxiety and depression symptoms, offering dynamic, evidence-based mental wellness support tailored to diverse needs. However, there is a need to evaluate its feasibility, acceptability, and preliminary effectiveness in a real-world setting.

The purpose of this study was to explore how college students interact with Wayhaven’s AI-powered mental wellness chatbot and its feasibility for improving mental wellness among college students with elevated depression or anxiety symptoms. This study aimed to: (1) determine the acceptability of Wayhaven; (2) determine the preliminary effectiveness of Wayhaven in improving mental health symptoms among students, including anxiety, depression, hopelessness, agency, and self-efficacy; and (3) explore student experiences and perceptions of Wayhaven. In this study, we use engagement (ie, actual use of the app) and participant satisfaction (ie, postuse evaluation) as proxies for acceptability.

## Methods

### Recruitment

Participants were recruited from a public university in New Jersey. Students learned about the study through an email newsletter that lists on-campus research opportunities that were sent to all students. The email provided information about the study and a link to a brief web-based survey to assess eligibility. After providing informed consent, participants were directed to take the screening survey to determine eligibility for the study. To be included, participants need to: (1) be aged 18 years or older; (2) be a student enrolled in the participating university; (3) live in the United States; (4) read and understand English; and (5) have elevated depression (Patient Health Questionnaire-2 [PHQ-2] [15] score ≥3) or anxiety symptoms (Generalized Anxiety Disorder 2 [GAD-2; [16] score ≥3).

### Procedures

Eligible participants were asked to complete a preintervention survey and to spend at least 5 minutes during their first Wayhaven conversation. At the end of the first session, they were directed to complete a postintervention survey. Following this initial conversation, participants continued to have access to Wayhaven for 1 week and were encouraged to engage with the AI mental wellness chatbot as much as needed. Participants received a final follow-up survey 1 week after enrollment. Each survey took 30‐45 minutes to complete. Participants were recruited from September 20 to September 25, 2024. Recruitment was closed once 40 students completed the study (ie, all assessments and at least one conversation). After completing the study, students were granted unlimited access to Wayhaven.

### Ethical Considerations

The Montclair State University Institutional Review Board approved this study (IRB-FY24-25-3875). All participants provided informed consent prior to particpating and were informed that they could to opt out of the study at any time. Participants received a US $20 gift card for completing each survey. All participant data was de-identified.

### Wayhaven AI Mental Wellness Coach

Participants were given access to Wayhaven upon study enrollment. Wayhaven is a generative AI-powered mental wellness chatbot, designed to deliver brief, evidence-based text conversations that support college students’ mental wellness needs. These conversations are rooted in CBT, dialectical behavior therapy, acceptance and commitment therapy, and mindfulness-based practices, developed in collaboration with mental health experts, including psychologists and mental health counselors. Student feedback has also been incorporated into Wayhaven’s working model. Specifically, before initiating this study, 74 students across 8 college campuses tested Wayhaven and responded to a brief open-ended survey eliciting feedback on the app. Students were also asked to participate in 45-minute focus groups (3‐6 students per group) where they completed design thinking sessions related to a particular feature or conversation flow change in the app. Six focus groups were conducted prior to the study.

Wayhaven aims to provide comprehensive mental wellness support to college students by offering tailored psychoeducational content and emotional support resources. The AI is designed to replicate empathic responses and offer specific techniques to address emotions or concerns shared by users. For example, a user expressing stress might first be validated and supported by the coach’s comments and then guided through a relaxation technique, ensuring a responsive and meaningful experience. Before starting a conversation, users select a coach persona that aligns with their preferences, such as a first-generation Latinx student or a university professor. This user-centered approach enhances engagement and flexibility, allowing users to switch between coaches while maintaining comprehensive support. Wayhaven also integrates university-specific context, such as details about campus resources, student clubs, and upcoming events, to ensure users have quick access to services available on their campus. By combining evidence-based practices with personalized AI interactions, Wayhaven not only addresses mental wellness concerns but also amplifies the utility of a school’s existing support infrastructure.

**Figure 1.**
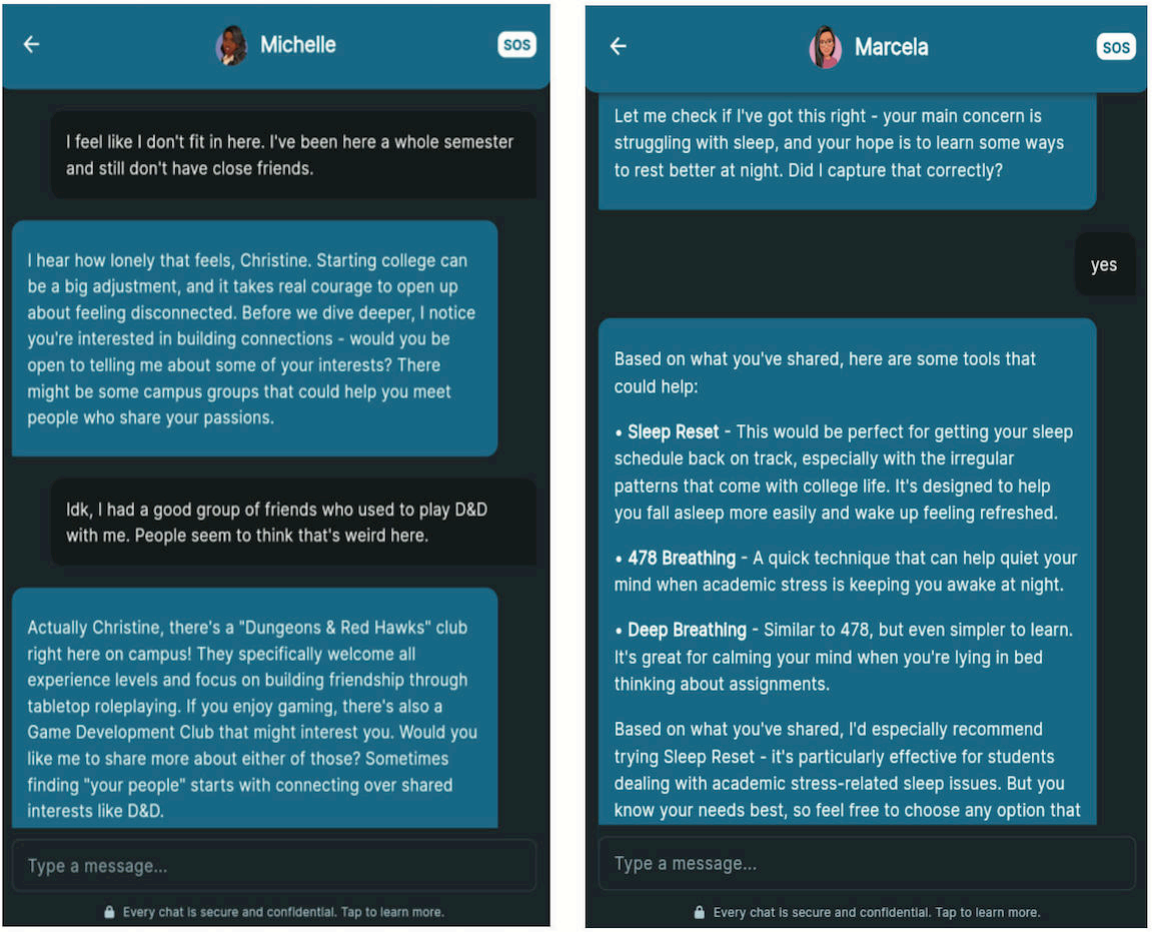
Example student conversations with the Wayhaven AI mental wellness coach. AI: artificial intelligence.

The user journey begins with account creation and onboarding, where participants provide demographic information and choose an AI mental wellness coach. Participants are able to choose from four options: Marcela (Latinx college student), Michelle (Black life coach), Professor Wilson (White college professor), and Coach Thompson (Black sports coach). Conversations follow a structured single-session intervention format, starting with the identification of a concern and a primary goal. The AI mental wellness chatbot then offers evidence-based tools, helps the user practice selected techniques, and collaborates to create an actionable plan. Each session concludes with a summary, and users can return for on-demand support at any time. To promote continued engagement, Wayhaven also sends push notifications to check in on users, ensuring ongoing and adaptive support. Figure 1 shows example conversations with the AI mental wellness coaches.

When creating an account, users are also informed that whenever they use Wayhaven, they will be chatting with an AI and not a real person, and that Wayhaven is not designed to address crises or substitute professional mental health care. Users seeking mental health services or reporting elevated distress are directed to campus-specific resources, such as the college counseling center. The app also features an “SOS” button, which allows users to connect to the Suicide and Crisis Lifeline (988) or the Crisis Text Line directly from the app. This feature appears at the top of each screen a user sees.

### Measures

#### Overview

In addition to participant demographics, the preintervention survey included measures of anxiety, depression, hopelessness, agency, self-efficacy, and well-being. The postintervention survey included measures of anxiety, depression, hopelessness, agency, self-efficacy, and satisfaction. The final follow-up survey included all of the aforementioned mental health outcomes and satisfaction measures.

#### Screening Survey

To determine eligibility for the study, students were asked to indicate whether they were currently enrolled in the participating university, aged 18 years or older, currently living in the United States, and could read and understand English. Participants also completed the PHQ-2 [15] and the GAD-2 [16] to assess depression and anxiety symptoms, respectively.

#### Demographics

In the preintervention survey, participants were asked to report their age, sex assigned at birth, gender identity, sexual orientation, race or ethnicity, year in school, enrollment status, where they currently live, financial aid status, if they were a first-generation college student, if they were working outside of attending school, and whether they were currently receiving mental health treatment.

#### Anxiety

The Generalized Anxiety Disorder-7 (GAD-7) [17] was used to assess generalized anxiety symptoms at baseline, postintervention, and 1-week follow-up. Time periods for this measure were adjusted such that there was no overlap prior to using the AI chatbot, during the 1-week use period, and after the 1-week use period. Participants reported how often during the past 2 weeks they were bothered by seven anxiety symptoms (eg, feeling nervous, anxious, or “on edge”) from 0=not at all to 3=every day. Scale scores were computed as the sum of all items. Cronbach α for this measure in this study was 0.74.

#### Depression

Depression symptoms were assessed using the Patient Health Questionnaire (PHQ-8) [18], an 8-item self-report measure that evaluates depressive symptoms within the previous 2 weeks, at baseline, postintervention, and 1-week follow-up. Time periods for this measure were adjusted such that there was no overlap prior to using the AI chatbot, during the 1-week use period, and after the 1-week use period. Participant responses ranged from 0=not at all to 3=nearly every day. Scale scores were computed as the sum of all items. Cronbach α for this measure in this study was 0.79.

#### Hopelessness

A modified version of the Beck Hopelessness Scale [19,20] was used to assess participants’ feelings of hopelessness. This scale consists of 4 items, including 3 items from the original measure (eg, “My future seems dark to me”) and 1 item from the Beck Depression Inventory [21] (eg, “I feel that the future is hopeless and that things cannot improve”). These 4 items have been found to correlate highly with the original Beck Hopelessness Scale and demonstrate excellent internal consistency [22-24]. Participant responses ranged from 0=absolutely disagree to 3=absolutely agree. Scale scores were computed as the sum of all items. Cronbach α for this measure in this study was 0.87.

#### Agency

The agency subscale of the State of Hope Scale [25] was used to assess agency or participants’ beliefs in their ability to initiate and sustain goals or actions. This subscale consists of 3 items (eg, “At the present time, I am energetically pursuing my goals”) measuring agency for goals. Participants rated how true each item was for them using an 8-point Likert scale, going from 1=definitely false to 8=definitely true. This measure has been found to be valid in previous research examining single-session interventions among adults [22]. Cronbach α for this measure in this study was 0.79.

#### Self-Efficacy

A modified version of the General Self-Efficacy Scale [26] was used to examine participants’ self-efficacy or the belief that one is able to control challenging environmental demands by taking adaptive action. This scale consists of 6 items from the original measure (eg, “It is easy for me to stick to my aims and accomplish my goals”). Participant responses ranged from 1=not at all to 4=exactly true. This scale has been found to be valid and reliable [26]. Cronbach α for this measure in this study was 0.81.

#### Well-Being

The Short Warwick-Edinburgh Mental Wellbeing Scale [27] was used to assess both subjective and psychological well-being. Participants completed this measure at postintervention and follow-up. Participants were asked to indicate how frequently they had experienced the feelings described in each of the 7 items on the Short Warwick-Edinburgh Mental Wellbeing Scale over the past 2 weeks (eg, “I’ve been feeling optimistic about the future”). Responses ranged from 1=none of the time to 5=all of the time. This measure has been found to be valid and reliable among college student samples [28]. Cronbach α for this measure in this study was 0.68.

Participants were also asked to rate their mental wellness following their use of Wayhaven via one item developed by the study investigators. Participants were first provided with the following definition of mental wellness: “Mental wellness refers to our ability to thrive emotionally and psychologically (eg, feeling in control of life, able to manage stress or emotions, positive view of self, and knowing how to find support).” They were then asked to rate the extent to which their mental wellness changed compared to before their session with the AI mental wellness coach. Response options ranged from 1=much worse to 5=much better. Participants completed the single item at postintervention.

#### Engagement

Engagement with Wayhaven was assessed using data acquired through the app on use and engagement with the intervention, including unique sessions, number of messages sent, and time spent per session.

#### Satisfaction

Participant satisfaction with Wayhaven was assessed via 14 questions developed by the study investigators that were informed by the Bowen et al [29] framework. Specifically, 5 items examine approval with Wayhaven (eg, “I was satisfied with my experience using Wayhaven”), 4 items assessed demand (eg, “I would choose to use Wayhaven over other mental wellness resources available to me, such as the college counseling center”), 3 items measured practicality (eg, “Using Wayhaven fit easily into my daily routine”), and 1 item was used to assess implementation (eg, “Wayhaven worked as intended without technical issues”). Participants were also asked to rate how engaging they found interactions with the Wayhaven AI mental wellness coach. Responses to this item ranged from 1=not at all to 5=extremely engaging. The list of items is provided in the Results section.

### Statistical Analysis

Analyses were conducted using SPSS (version 28; IBM Corp). Descriptive statistics were used to summarize participant characteristics, primary outcomes, app use and engagement, and satisfaction with the AI coach. Univariate outliers were identified as having *z* scores greater than 3. When outliers were present, univariate analyses were run without outliers. Multilevel modeling (MLM) was used to examine the main study hypotheses. MLM was used because (1) it allows the inclusion of all participants, regardless of missing data, while efficiently handling missing data; and (2) it is considered the preferred method to analyze longitudinal psychiatric data [30]. The linear mixed-effects models procedure of SPSS 28 was used for these analyses. For depression on the PHQ-8, a two-level MLM model using maximum likelihood estimation was computed. At Level 1, depression symptoms varied within participants over time as a function of a person-specific growth curve. This level tested for time effects, estimating the change in depression across the pre, postintervention, and follow-up assessment points. Time was coded such that the intercept reflected the initial level of depression at preintervention. Similar MLM models were computed for anxiety, hopelessness, self-efficacy, and agency. For each MLM model, we also computed the proportional reduction explained at the individual level, conceptualized as a change in pseudo *R*^2^ when the time was added to the empty model (ie, random intercept-only model without any predictors). We used the following Snijders and Bosker [31] formula: *R*^2^=1−(*σ*^2^_F_ + *τ*^2^_F_/ *σ*^2^_E_ + *τ*^2^_E_), where *σ*^2^_F_ represents the level-one random error variance for the full model with time; *τ*^2^_F_ represents the level-two random error variance for the full model; *σ*^2^_E_ represents the level-one random error variance for the empty model; and *τ*^2^_E_ represents the level-two random error variance for the empty model. The effect size measure related to the variance explained for the overall model is *⨍*^2^=*R^2^*/1*−R^2^* [32,33]. Guidelines for interpretation of *⨍*^2^ indicate that 0.02 is a small effect, 0.15 is a medium effect, and 0.35 is a large effect [34]. Finally, for outcome measures assessed at only two time points, such as well-being, 2-tailed paired samples *t* tests were used to examine within-participant change. Cohen *d* was calculated as a measure of effect size, with values of 0.2, 0.5, and 0.8 interpreted as small, medium, and large effects, respectively [32].

## Results

### Preliminary Analyses

Of the 113 students who were eligible to participate, 50 students completed the pre- and postintervention surveys and at least 1 session with the app, and 41 students completed the 1-week follow-up survey. No significant differences were found between those who completed the follow-up survey and those who did not in terms of gender or baseline severity of depression or anxiety symptoms. A significant difference was found in terms of age (*t*_48_= 2.42; *P*=.02), such that participants who did not complete the follow-up survey were significantly older (mean age 25, SD 6.41 y) than participants who completed the follow-up survey (mean age 21.4, SD 3.51 y). However, this difference is relatively modest and may not represent a clinically meaningful distinction among this age group. Accuracy checks assessing the range of summed scores (minimum and maximum scores) for each scale indicated that participants accurately completed the scales.

Of the 50 students who completed at least one session, 90% (n=45) were female. The mean age of the sample was 22.12 (SD 4.42) years. About 46% (n=23) self-identified as Latinx, 34% (n=17) non-Latinx White, 8% (n=4) non-Latinx Black, 8% (n=4) non-Latinx Asian, and 4% (n=2) multiracial. Most students were seniors (n=14, 28%). Half the students self-identified as heterosexual or straight (n=25). Most students were enrolled full-time (n=46, 92%), and 58% (n=29) were commuters or lived off-campus. Most students (n=36, 72%) also reported receiving federal or state financial assistance for their college education and being first-generation college students (n=29, 58%; see Table 1 for demographic characteristics of the sample).

The mean baseline PHQ-8 and GAD-7 scores were 13.50 (SD 5.06; range 4‐21) and 14 (SD 4.03; range 3‐23), respectively, with 80% (n=40) of students scoring above the clinical cutoff on the PHQ-8 (score ≥10) and 88% (n=44) on GAD-7 (score ≥10). The average hopelessness score was 5.38 (SD 2.67; range 0‐12). The mean agency score was 14.14 (SD 5.29; range 3‐23), self-efficacy was 15.98 (SD 3.98; range 4‐23), and wellness was 19.50 (SD 4.52; range 4‐29). Table 2 presents the mean score for each outcome of interest across the three study time points.

**Table 1.** Demographic characteristics.

Variable	Value, n (%)
Sex^a^	
Female	45 (90)
Male	5 (10)
Gender^a^	
Female	40 (80)
Male	4 (8)
Transgender man or transman	1 (2)
Genderqueer	4 (8)
Other	1 (2)
Sexual orientation	
Straight or heterosexual	25 (50)
Bisexual	11 (22)
Gay or lesbian	4 (8)
Pansexual	3 (6)
Queer	3 (6)
Asexual	2 (4)
Other	2 (4)
Race or ethnicity	
Latinx	23 (46)
Non-Latinx White	17 (34)
Non-Latinx Black	4 (8)
Non-Latinx Asian	4 (8)
Multiracial	2 (4)
Year in school	
Freshman	8 (16)
Sophomore	10 (20)
Junior	11 (22)
Senior	14 (28)
Graduate student	7 (14)
Enrollment status	
Full-time	46 (92)
Part-time	4 (8)
Current living	
On-campus (ie, university housing)	21 (42)
Off-campus (ie, nonuniversity housing near campus)	6 (12)
Commuter (ie, permanent home or residence a significant distance from campus)	23 (46)
Receiving financial aid	
Yes	36 (72)
No	14 (28)
First-generation college student	
Yes	29 (58)
No	21 (42)
Working outside of attending school	
Yes (employed part-time)	24 (48)
Yes (employed full-time)	5 (10)
No	19 (38)
Prefer not to disclose	2 (4)
Current mental health treatment	
Yes	18 (36)
No	27 (54)
Prefer not to disclose	5 (10)

a Both sex and gender are reported to reflect biological and self-identified dimensions, respectively. While the distributions are similar in this sample, we report both for transparency and inclusivity.

**Table 2.** Means and SDs for outcomes across assessments.

Variable	Pre (n=50), mean (SD)	Post^a^ (n=50), mean (SD)	1-week follow-up (n=41), mean (SD)
Depression (PHQ-8)^b^	13.50 (5.06)	13.46 (2.92)	10.17 (5.31)
Anxiety (GAD-7)^c^	14.00 (4.03)	11.14 (4.08)	9.76 (5.64)
Hopelessness (BHS)^d^	5.38 (2.67)	4.18 (2.65)	4.27 (2.71)
Agency (SHS)^e^	14.14 (5.29)	15.52 (4.98)	15.24 (5.34)
Self-Efficacy (GSE)^f^	15.98 (3.98)	16.84 (4.07)	16.95 (3.82)
Well-Being (SWEMWBS)^g^	10.50 (4.52)	N/A^h^	21.66 (5.05)

a Postassessment occurred after the first session.

b PHQ-8: Patient Health Questionnaire-8.

c GAD-7: Generalized Anxiety Disorder-7.

d BHS: Beck Hopelessness Scale.

e SHS: State of Hope Scale.

f GSE: General Self-Efficacy Scale.

g SWEMWBS: Short Warwick-Edinburgh Mental Well-being Scale.

h Not applicable.

The mean number of sessions attended was 2.02 (SD 1.86; range 1‐10), suggesting that, on average, students completed around two sessions over the 1-week period with the AI mental health wellness coach. Of the four coach characters offered, the mean number of coaches used was 1.14 (SD 0.45; range 1‐3), which may suggest that students often opted to use the same coach character across sessions. Specifically, Michelle was selected a total of 19 times, Marcela was selected a total of 20 times, Professor Wilson was selected a total of 16 times, and Coach Thompson was selected a total of 2 times. On average, students used 1.1 (SD 1.13; range 0‐5) CBT-based skills during their conversations (ie, grounding techniques, mindfulness exercises, and exploring and challenging cognitive distortions). One univariate outlier was identified with regard to minutes spent per session (ie, *z* score above 3). After removing this participant from the analysis, the average minutes students spent in each session was 15.43 (SD 10.11; range 3.18‐53.48). Two outliers were also present with regard to message count. The average message count was 29.08 (SD 22.43; range 5‐115) after these two students were removed from the analysis. Finally, two outliers were identified for students’ response time. The students’ mean response time to each AI-generated response was 7.33 (SD 24.55; range 0.13‐143.37) minutes once these cases were excluded.

### Improvement in Outcome Scores

A linear model with random slopes and intercepts revealed that students’ GAD-7 scores decreased significantly from preintervention to 1-week follow-up (β=−2.15; *P*<.001). About 14% of the variance in anxiety was explained by time (*R*^2^=0.13; *⨍*^2^=0.14). Similarly, there was a significant reduction in students’ PHQ-8 (β=−1.62; *P*<.001) and hopelessness scores (β=−.64; *P*<.001), with time accounting for 8% and 3% of the variance, respectively (*R*^2^=0.07, *⨍*^2^=0.08; *R*^2^=0.02, *⨍*^2^=0.03, respectively). With regard to agency and efficacy, students’ scores significantly increased from preintervention to follow-up (β=.64, *P*=.32; β=.53, *P*=.02, respectively). However, time accounted for less than 1% of the variance in agency (*R*^2^=0.007; *⨍*^2^=0.007) and efficacy (*R*^2^=0.009 *⨍*^2^=0.009). See Table 3 and Multimedia Appendix 1 for results of MLM analyses. To examine changes in well-being, a 2-tailed paired samples *t* test was computed. Students’ well-being scores significantly increased from preintervention (mean 18.98, SD 4.35) to follow-up (mean 21.66, SD 0.79; *t*_40_=2.90; *P*=.006, *d*=0.45). Relatedly, at postintervention, 62% (n=31) of students reported that their mental wellness was better or much better after one session with Wayhaven. As a sensitivity analysis, the current mental health treatment status was added as a covariate to the linear mixed models. This variable did not meaningfully affect the pattern or significance of the results. Thus, it was not included in the final models reported here.

**Table 3.** Parameters for time predicting change in outcomes over 1 week post intervention.

Outcome and predictor	Estimate	SE	T ratio	95% CI	*⨍* ^2a^
Anxiety					0.14
Intercept	13.76	0.59	23.34^b^	12.60 to 14.93	
Time	−2.15	0.47	−4.60^b^	−3.08 to −1.22	
Depression					0.08
Intercept	14.03	0.60	23.50^b^	12.84 to 15.21	
Time	−1.62	0.43	−3.74^b^	−2.47 to −0.76	
Hopelessness					0.03
Intercept	5.19	0.37	13.99^b^	4.45 to 5.95	
Time	−.64	0.16	−4.09^b^	−0.94 to −0.33	
Agency					0.007
Intercept	14.39	0.72	19.95^b^	12.95 to 15.83	
Time	.64	0.30	2.17^c^	0.06 to 1.23	
Self-efficacy					0.009
Intercept	16.09	0.55	29.37^b^	14.99 to 17.18	
Time	.54	0.23	2.35^c^	0.08 to 0.99	

a *⨍*2: effect size.

b *P*<.001.

c *P*<.05.

### Satisfaction With the App

Immediately after using Wayhaven (ie, at postintervention), participants generally endorsed that they found Wayhaven to be acceptable. Specifically, 90% (n=45) of participants agreed that Wayhaven was easy to use, and 74% (n=37) of participants indicated that they were satisfied with their experience with Wayhaven. Most participants (n=36, 72%) also agreed that they would use Wayhaven again in the future. The majority of students (n=42, 84%) participants also agreed that there is a need for a resource like Wayhaven among college students. Many students also reported finding the app quite engaging (n=26, 53%).

At 1-week follow-up, there was a slight decrease in students’ satisfaction, with 65% (27/41) of participants indicating that they were satisfied with their experience using Wayhaven. Additionally, 59% (24/41) of participants indicated that they would use Wayhaven again in the future. Despite these shifts, 68% (27/41) of participants noted that they would recommend Wayhaven to a friend who may be experiencing similar mental wellness concerns. Participants also endorsed the strong feasibility of Wayhaven, such that 80% (33/41) of participants felt that Wayhaven fit easily into their daily routine (see Table 4 for detailed frequency of student responses to satisfaction questions at postintervention and follow-up). More than half of the students also continued to find the app quite engaging (22/41, 56%).

**Table 4.** Frequency of student responses to items on satisfaction survey (items 1‐13).

Item and time point		Strongly disagree, n (%)	Disagree, n (%)	Neither agree nor disagree, n (%)	Agree, n (%)	Strongly agree, n (%)	Prefer not to say, n (%)
I found Wayhaven easy to use							
Postintervention		1 (2)	0 (0)	3 (6)	17 (34)	28 (56)	1 (2)
Follow-up		1 (2)	1 (2)	1 (2)	20 (49)	18 (44)	0 (0)
I was satisfied with my experience using Wayhaven							
Postintervention		1 (2)	4 (8)	8 (16)	22 (44)	15 (30)	0 (0)
Follow-up		1 (2)	5 (12)	8 (20)	13 (32)	14 (34)	0 (0)
I would use Wayhaven again in the future							
Postintervention		2 (4)	3 (6)	9 (18)	20 (40)	16 (32)	0 (0)
Follow-up		3 (7)	7 (17)	7 (17)	7 (17)	17 (42)	0 (0)
Wayhaven fits well with how I prefer to address my mental wellness concerns							
Postintervention		4 (8)	4 (8)	18 (36)	14 (28)	10 (20)	0 (0)
Follow-up		6 (15)	6 (15)	13 (32)	7 (17)	9 (22)	0 (0)
People close to me agree or would agree with me using Wayhaven							
Postintervention		6 (12)	3 (6)	14 (28)	18 (36)	9 (18)	0 (0)
Follow-up		3 (7)	4 (10)	7 (17)	18 (44)	9 (22)	0 (0)
I would recommend Wayhaven to a friend experiencing similar mental wellness concerns							
Postintervention		4 (8)	4 (8)	6 (12)	24 (48)	11 (22)	1 (2)
Follow-up		4 (10)	3 (7)	6 (15)	16 (39)	11 (27)	1 (2)
I would choose to use Wayhaven over other mental wellness resources available to me, such as the college counseling center							
Postintervention		8 (16)	10 (20)	13 (26)	11 (22)	7 (14)	1 (2)
Follow-up		9 (22)	12 (29)	5 (12)	4 (10)	10 (24)	1 (2)
I intend to continue using Wayhaven							
Postintervention		3 (6)	6 (12)	7 (14)	20 (40)	14 (28)	0 (0)
Follow-up		5 (12)	3 (7)	7 (17)	14 (34)	11 (27)	1 (2)
I believe there is a need for a resource like Wayhaven among college students							
Postintervention		2 (4)	1 (2)	4 (8)	17 (34)	25 (50)	1 (2)
Follow-up		2 (5)	1 (2)	2 (5)	14 (34)	22 (54)	0 (0)
I was able to use Wayhaven without requiring any additional resources or support							
Postintervention		2 (4)	2 (4)	5 (10)	11 (22)	30 (60)	0 (0)
Follow-up		2 (5)	1 (2)	3 (7)	15 (37)	20 (49)	0 (0)
Using Wayhaven fits easily into my daily routine							
Postintervention		1 (2)	1 (2)	6 (12)	24 (48)	17 (34)	1 (2)
Follow-up		1 (2)	0 (0)	7 (17)	14 (34)	19 (46)	0 (0)
The advice that Wayhaven provided was practical and easy for me to carry out in my daily life							
Postintervention		2 (4)	3 (6)	6 (12)	23 (46)	15 (30)	1 (2)
Follow-up		1 (2)	3 (7)	10 (24)	14 (34)	13 (32)	0 (0)
Wayhaven worked as intended without technical issues							
Postintervention		0 (0)	3 (6)	4 (8)	16 (32)	25 (50)	2 (4)
Follow-up		1 (2)	4 (10)	3 (7)	13 (32)	19 (46)	1 (2)

## Discussion

### Principal Results

The purpose of this study was to explore how college students interact with Wayhaven’s AI-powered mental wellness chatbot and its feasibility for improving mental wellness among college students with elevated depression or anxiety symptoms. Specifically, this study sought to assess the feasibility and acceptability (ie, engagement and satisfaction) of using Wayhaven with students with elevated depression or anxiety symptoms, as well as whether app use was linked with improvement across several mental health outcomes over a 1-week period. Most participants self-identified as racially and ethnically minoritized and were experiencing clinically elevated depression or anxiety symptoms. Overall, Wayhaven use was associated with a significant decrease in depression, anxiety, and hopelessness from preintervention to follow-up. Wayhaven use was also related to a significant increase in agency, efficacy, and well-being across the study period. Additionally, students reported being satisfied with Wayhaven and seeing the app as a new important resource for college students.

More than half of the participants scored above the clinical cutoff on the PHQ-8 and GAD-7, which is in line with previous research documenting elevated rates of internalizing symptoms among college students [2]. It is clear that there is a need for increased access to efficacious and equitable mental health resources for college students, particularly those self-identifying as racially and ethnically minoritized [35,36]. Of note, 66% (n=33) of students in our sample were racially and ethnically minoritized. DMHIs may be particularly appealing to racially and ethnically minoritized students who are more likely than White students to indicate a preference for web-based treatment versus in-person therapy [37,38]. Our findings suggest that Wayhaven may be a viable mental health resource to such populations, given the reduction in depression, anxiety, and hopelessness scores in a relatively short period of time.

Results also suggest that the majority of students were satisfied with their experience using Wayhaven. Most students felt that there was a need for a resource like Wayhaven among college students and that they would recommend it to their peers. With regard to the coach personalities offered, most students opted to use the same coach persona across sessions and selected Marcela, the Latinx college student, most frequently. This preference among students aligns with previous research suggesting that college students tend to favor seeking help from informal sources, like peers [39]. In addition to rating the app as engaging, students completed two sessions on the app on average across the 1-week study period, with the average message count across sessions being around 29 (SD 22.43). The number of messages students exchanged is comparable to those reported by He et al [40], who tested a cognitive behavioral therapy−based mental health chatbot, XiaoE, for young adults with depressive symptoms. They found that the average number of messages exchanged with XiaoE was 25.54 (SD 26.25) daily across a 1-week period. It should be noted that participants in this study were instructed and prompted to engage with XiaoE daily, whereas students in this study were asked to use Wayhaven as needed. It is unclear whether user engagement (ie, sessions and messages) would be higher if students were asked to use the tool daily. Taken together, these data suggest that Wayhaven appears to be engaging, acceptable, and feasible for college students with elevated depression or anxiety symptoms.

### Limitations

While these preliminary findings support the applicability of Wayhaven in college populations, this study is not without its limitations; caution should be used when considering the generalizability of the findings. This was a small, uncontrolled pilot study, and we did not include a control group. Therefore, we cannot infer that the observed symptom improvements were a result of the intervention. However, our goal was not to conduct a randomized controlled trial (RCT); instead, our aim was to test the feasibility and acceptability of a novel AI mental wellness chatbot in a real-world setting. Testing an intervention’s feasibility and acceptability via an open trial is an important first step prior to an RCT [41]. As noted above, this research should be followed up with a larger RCT comparing Wayhaven to an active control condition matched by age and sex and a longer follow-up period. The use of self-report assessments is also limited in that participants may have under- or overreported the severity of their symptoms. Importantly, self-report questionnaires of depression and anxiety symptoms have been found to be moderately to strongly correlated with clinician-rated scales, and previous work using these measures has proven to be clinically impactful [42,43]. The majority of participants were also female, possibly limiting generalizability to male participants. Additionally, our sample was racially and ethnically diverse, participants were obtained from a single public university in New Jersey, and results may not be generalizable to other areas of the United States.

Although Wayhaven use was associated with a statistically significant improvement in outcome scores across time, the magnitude of these effects was small. The observed effect sizes were strongest for anxiety, followed by depression, which were our main clinical outcomes. These findings are consistent with prior research on brief interventions that suggests such programs have the strongest effects on anxiety symptoms [44,45]. Researchers have noted that categorizing effect sizes as “small,” “medium,” or “large” is not inherently meaningful without a contextual frame of reference [46]. In public health, even small effects can yield a substantial impact when scaled across populations. Small shifts in mean scores can disproportionately affect the extreme end of a population distribution and drive higher rates of clinical referrals, as illustrated by the surge in mental health service use among youth during the COVID-19 pandemic despite only a modest increase in mental health outcome scores [47]. Moreover, brief interventions with small average effects may still yield substantial population-level benefits by offering a low-cost means to expand the reach and impact of mental health services, particularly for individuals in need, like college students who may be reluctant to seek higher-intensity support [45,48]. Nevertheless, future research should explore ways to strengthen and sustain the impact of Wayhaven, such as incorporating booster sessions or embedding the intervention within a broader stepped-care framework.

### Comparison With Prior Work

Our findings mirror previous research on the effectiveness of AI chatbots in reducing depression and anxiety symptoms among college students [13,14,41]. However, in contrast to previous studies testing scripted chatbots, Wayhaven’s generative AI-powered mental wellness chatbot offered students personalized, context-sensitive support by integrating demographic information, university-specific resources, and a customized AI coach persona. Given our preliminary findings, future research should focus on comparing the effectiveness of Wayhaven to an active control condition, such as a mental health app or internet-based intervention, and among a larger sample of students. If proven effective, Wayhaven can serve as a lower-intensity intervention that college counseling centers can offer to students presenting with subclinical or acute depression or anxiety symptoms, which can help free in-person services to students experiencing more severe symptoms or distress.

### Conclusions

Despite limitations, results revealed that Wayhaven led to an improvement in student mental well-being outcomes (ie, depression, anxiety, hopelessness, agency, efficacy, and well-being) and students were satisfied with the tool. Wayhaven may be a promising resource to deliver brief, evidence-based text conversations to support college students’ mental wellness needs. Future research should examine the effectiveness of Wayhaven by comparing it to an active control condition and including a larger student sample. With the already overwhelming and unmet mental health needs of this population, a DMHI like Wayhaven may offer a pathway to free, accessible, and equitable mental wellness care.

## Supplementary material

10.2196/71923 Multimedia Appendix 1 Multilevel models for time predicting change in outcomes over 1 week post intervention.

## Abbreviations

AI: artificial intelligence
CBT: cognitive behavioral therapy
DMHI: digital mental health intervention
GAD: Generalized Anxiety Disorder
MLM: multilevel modeling
PHQ: Patient Health Questionnaire
RCT: randomized controlled trial

## References

[1] McLafferty M, Brown N, McHugh R (2021). Depression, anxiety and suicidal behaviour among college students: comparisons pre-COVID-19 and during the pandemic. Psychiatry Res Commun.

[2] Wang X, Hegde S, Son C, Keller B, Smith A, Sasangohar F (2020). Investigating mental health of US college students during the COVID-19 pandemic: cross-sectional survey study. J Med Internet Res.

[3] Lipson SK, Diaz Y, Davis J, Eisenberg D (2023). Mental health among first-generation college students: findings from the National Healthy Minds Study, 2018-2021. Cogent Ment Health.

[4] Gorman B, Hargraves L, Huynh S, Parker S, Wiafe B (2020). Early findings from Transitions I: mental health, stress and health-related behaviors. https://transitions.web.unc.edu/wp-content/uploads/sites/22616/2020/10/Transitions-Wave-One-Report.pdf.

[5] Walls KL (2023). Navigating a new model for therapy at a university counseling center: a case example. Psychol Serv.

[6] Gulliver A, Griffiths KM, Christensen H (2010). Perceived barriers and facilitators to mental health help-seeking in young people: a systematic review. BMC Psychiatry.

[7] Vidourek RA, King KA, Nabors LA, Merianos AL (2014). Students’ benefits and barriers to mental health help-seeking. Health Psychol Behav Med.

[8] Lipschitz JM, Pike CK, Hogan TP, Murphy SA, Burdick KE (2023). The engagement problem: a review of engagement with digital mental health interventions and recommendations for a path forward. Curr Treat Options Psychiatry.

[9] Boucher EM, Harake NR, Ward HE (2021). Artificially intelligent chatbots in digital mental health interventions: a review. Expert Rev Med Devices.

[10] Koulouri T, Macredie RD, Olakitan D (2022). Chatbots to support young adults’ mental health: an exploratory study of acceptability. ACM Trans Interact Intell Syst.

[11] Kuhail MA, Alturki N, Thomas J, Alkhalifa AK (2024). Human vs. AI counseling: college students’ perspectives. Comput Hum Behav Rep.

[12] Rasouli S, Ghafurian M, Nilsen ES, Dautenhahn K (2024). University students’ opinions on using intelligent agents to cope with stress and anxiety in social situations. Comput Human Behav.

[13] Fitzpatrick KK, Darcy A, Vierhile M (2017). Delivering cognitive behavior therapy to young adults with symptoms of depression and anxiety using a fully automated conversational agent (Woebot): a randomized controlled trial. JMIR Ment Health.

[14] Klos MC, Escoredo M, Joerin A, Lemos VN, Rauws M, Bunge EL (2021). Artificial intelligence-based chatbot for anxiety and depression in university students: pilot randomized controlled trial. JMIR Form Res.

[15] Kroenke K, Spitzer RL, Williams JBW (2003). The Patient Health Questionnaire-2: validity of a two-item depression screener. Med Care.

[16] Kroenke K, Spitzer RL, Williams JBW, Monahan PO, Löwe B (2007). Anxiety disorders in primary care: prevalence, impairment, comorbidity, and detection. Ann Intern Med.

[17] Spitzer RL, Kroenke K, Williams JBW, Löwe B (2006). A brief measure for assessing generalized anxiety disorder: the GAD-7. Arch Intern Med.

[18] Kroenke K, Strine TW, Spitzer RL, Williams JBW, Berry JT, Mokdad AH (2009). The PHQ-8 as a measure of current depression in the general population. J Affect Disord.

[19] Beck AT, Weissman A, Lester D, Trexler L (1974). The measurement of pessimism: the hopelessness scale. J Consult Clin Psychol.

[20] Forintos DP, Rózsa S, Pilling J, Kopp M (2013). Proposal for a short version of the Beck Hopelessness Scale based on a national representative survey in Hungary. Community Ment Health J.

[21] Beck AT, Ward CH, Mendelson M, Mock J, Erbaugh J (1961). An inventory for measuring depression. Arch Gen Psychiatry.

[22] Schleider JL, Mullarkey MC, Fox KR (2022). A randomized trial of online single-session interventions for adolescent depression during COVID-19. Nat Hum Behav.

[23] Brott KH, Veilleux JC (2024). Examining state self-criticism and self-efficacy as factors underlying hopelessness and suicidal ideation. Suicide Life Threat Behav.

[24] Sung JY, Bugatti M, Vivian D, Schleider JL (2023). Evaluating a telehealth single-session consultation service for clients on psychotherapy wait-lists. Pract Innov (Wash D C).

[25] Snyder CR, Sympson SC, Ybasco FC, Borders TF, Babyak MA, Higgins RL (1996). Development and validation of the State Hope Scale. J Pers Soc Psychol.

[26] Romppel M, Herrmann-Lingen C, Wachter R (2013). A short form of the General Self-Efficacy Scale (GSE-6): development, psychometric properties and validity in an intercultural non-clinical sample and a sample of patients at risk for heart failure. Psychosoc Med.

[27] Stewart-Brown S, Tennant A, Tennant R, Platt S, Parkinson J, Weich S (2009). Internal construct validity of the Warwick-Edinburgh Mental Well-being Scale (WEMWBS): a Rasch analysis using data from the Scottish Health Education Population Survey. Health Qual Life Outcomes.

[28] Barbayannis G, Bandari M, Zheng X, Baquerizo H, Pecor KW, Ming X (2022). Academic stress and mental well-being in college students: correlations, affected groups, and COVID-19. Front Psychol.

[29] Bowen DJ, Kreuter M, Spring B (2009). How we design feasibility studies. Am J Prev Med.

[30] Seidel A, Presnell K, Rosenfield D (2009). Mediators in the dissonance eating disorder prevention program. Behav Res Ther.

[31] Snijders TAB, Bosker RJ (2012). Multilevel Analysis: An Introduction to Basic and Advanced Multilevel Modeling.

[32] Cohen J (1988). Statistical Power Analysis for the Behavioral Sciences.

[33] Lorah J (2018). Effect size measures for multilevel models: definition, interpretation, and TIMSS example. Large-scale Assess Educ.

[34] Cohen J (1992). A power primer. Psychol Bull.

[35] Lipson SK, Lattie EG, Eisenberg D (2019). Increased rates of mental health service utilization by U.S. college students: 10-year population-level trends (2007-2017). Psychiatr Serv.

[36] Miranda R, Soffer A, Polanco-Roman L, Wheeler A, Moore A (2015). Mental health treatment barriers among racial/ethnic minority versus white young adults 6 months after intake at a college counseling center. J Am Coll Health.

[37] Dunbar MS, Sontag-Padilla L, Kase CA, Seelam R, Stein BD (2018). Unmet mental health treatment need and attitudes toward online mental health services among community college students. Psychiatr Serv.

[38] Lungu A, Sun M (2016). Time for a change: college students’ preference for technology-mediated versus face-to-face help for emotional distress. Telemed e-Health.

[39] Eisenberg D, Hunt J, Speer N (2012). Help seeking for mental health on college campuses: review of evidence and next steps for research and practice. Harv Rev Psychiatry.

[40] He Y, Yang L, Zhu X (2022). Mental health chatbot for young adults with depressive symptoms during the COVID-19 pandemic: single-blind, three-arm randomized controlled trial. J Med Internet Res.

[41] Rounsaville BJ, Carroll KM, Onken LS (2001). A stage model of behavioral therapies research: getting started and moving on from stage I. Clin Psychol Sci Pract.

[42] Rush AJ, Carmody TJ, Ibrahim HM (2006). Comparison of self-report and clinician ratings on two inventories of depressive symptomatology. Psychiatr Serv.

[43] Uher R, Perlis RH, Placentino A (2012). Self-report and clinician-rated measures of depression severity: can one replace the other?.. Depress Anxiety.

[44] Cape J, Whittington C, Buszewicz M, Wallace P, Underwood L (2010). Brief psychological therapies for anxiety and depression in primary care: meta-analysis and meta-regression. BMC Med.

[45] Schleider JL, Zapata JP, Rapoport A (2025). Single-session interventions for mental health problems and service engagement: umbrella review of systematic reviews and meta-analyses. Annu Rev Clin Psychol.

[46] Funder DC, Ozer DJ (2019). Evaluating effect size in psychological research: sense and nonsense. Adv Methods Pract Psychol Sci.

[47] Carey EG, Ridler I, Ford TJ, Stringaris A (2023). Editorial perspective: when is a “small effect” actually large and impactful?. J Child Psychol Psychiatry.

[48] Greenberg MT, Abenavoli R (2017). Universal interventions: fully exploring their impacts and potential to produce population-level impacts. J Res Educ Eff.

