# Supplementary material for: Generative AI–Powered Mental Wellness Chatbot for College Student Mental Wellness: Open Trial
Source: JMIR Form Res. 2025 Jul 28;9:e71923. doi: 10.2196/71923 (PMC12303582; doi:10.2196/71923)
Supplement: Multimedia Appendix 1 [file formative-v9-e71923-s001.docx]

|  | | | | | | | | |
| --- | --- | --- | --- | --- | --- | --- | --- | --- |
| Outcome: Anxiety | | | | | | | | |
|  |  | | |  | | |  |  |
|  | Empty Model | | | Full Model | | |  |  |
| Predictor | Estimate | SE | 95% CI | Estimate | SE | 95% CI | *R^2^* | *⨍^2^* |
| Intercept | 11.75 | .41 | 10.94, 12.56 | 13.76 | .59 | 12.60, 14.93 | 0.13 | 0.14 |
| Time |  |  |  | -2.15 | .47 | -3.08, -1.22 |  |  |
| Within Person Variance, σ^2^ | 23.52 |  |  | 19.72 |  |  |  |  |
| Between Person Variance, τ^2^ | .00 |  |  | .83 |  |  |  |  |
| Outcome: Depression | | | | | | | | |
|  | Empty Model | | | Full Model | | |  |  |
| Predictor | Estimate | SE | 95% CI | Estimate | SE | 95% CI | *R^2^* | *⨍^2^* |
| Intercept | 12.52 | .44 | 11.64, 13.41 | 14.03 | .60 | 12.84, 15.21 | 0.07 | 0.08 |
| Time |  |  |  | -1.62 | .43 | -2.47, -.76 |  |  |
| Within Person Variance, σ^2^ | 19.19 |  |  | 16.66 |  |  |  |  |
| Between Person Variance, τ^2^ | 2.94 |  |  | 3.82 |  |  |  |  |
| Outcome: Hopelessness | | | | | | | | |
|  | Empty Model | | | Full Model | | |  |  |
| Predictor | Estimate | SE | 95% CI | Estimate | SE | 95% CI | *R^2^* | *⨍^2^* |
| Intercept | 4.61 | .34 | 3.93, 5.30 | 5.19 | .37 | 4.45, 5.95 | 0.02 | 0.03 |
| Time |  |  |  | -.64 | .16 | -.94, -.33 |  |  |
| Within Person Variance, σ^2^ | 2.50 |  |  | 2.11 |  |  |  |  |
| Between Person Variance, τ^2^ | 4.90 |  |  | 5.11 |  |  |  |  |
| Outcome: Agency | | | | | | | | |
|  | Empty Model | | | Full Model | | |  |  |
| Predictor | Estimate | SE | 95% CI | Estimate | SE | 95% CI | *R^2^* | *⨍^2^* |
| Intercept | 14.97 | .67 | 13.63, 16.31 | 14.39 | .72 | 12.95, 15.83 | 0.007 | 0.007 |
| Time |  |  |  | .64 | .30 | .06, 1.23 |  |  |
| Within Person Variance, σ^2^ | 7.99 |  |  | 7.59 |  |  |  |  |
| Between Person Variance, τ^2^ | 19.39 |  |  | 19.61 |  |  |  |  |
| Outcome: Self-Efficacy | | | | | | | | |
|  | Empty Model | | | Full Model | | |  |  |
| Predictor | Estimate | SE | 95% CI | Estimate | SE | 95% CI | *R^2^* | *⨍^2^* |
| Intercept | 16.58 | .51 | 15.56, 17.59 | 16.09 | .55 | 14.99, 17.18 | 0.009 | 0.009 |
| Time |  |  |  | .54 | .23 | .08, .99 |  |  |
| Within Person Variance, σ^2^ | 4.80 |  |  | 4.52 |  |  |  |  |
| Between Person Variance, τ^2^ | 11.07 |  |  | 11.20 |  |  |  |  |

Note: CI = Confidence interval; *R^2^* = Proportional reduction of explained variance; ⨍^2^ = Effect size related to variance explained
